# Examining intersectoral integration for malaria control programmes in an urban and a rural district in Ghana: a multinomial multilevel analysis

**DOI:** 10.5334/ijic.1061

**Published:** 2013-08-07

**Authors:** Nicodemus Osei Owusu, Bernard Baffour-Awuah, Fiifi Amoako Johnson, John Mohan, Nyovani J. Madise

**Affiliations:** School of Applied Health Sciences, Department of Nursing, Central University College, Tema, Ghana; Institute for Social Science Research, University of Queensland, Brisbane, Australia; Division of Social Statistics & Demography and Centre for Global Health, Population, Poverty and Policy (GHP3), Faculty of Social and Human Sciences, University of Southampton, Southampton, UK; Division of Sociology and Social Policy, Faculty of Social and Human Sciences, University of Southampton, Southampton, UK; Division of Social Statistics & Demography and Centre for Global Health, Population, Poverty and Policy (GHP3), Faculty of Social and Human Sciences, University of Southampton, Southampton, UK

**Keywords:** intersectoral integration, intersectoral collaboration, malaria control programmes, multilevel multinomial ordinal regression

## Abstract

**Background:**

Intersectoral integration is acknowledged to be essential for improving provision of health care and outcomes, yet it remains one of the main primary health care strategic challenges. Although this is well articulated in the literature, the factors that explain differentials in levels of intersectoral integration have not been systematically studied, particularly in low and middle-income countries. In this study, we examine the levels and determinants of intersectoral integration amongst institutions engaged in malaria control programmes in an urban (Kumasi Metropolitan) district and a rural (Ahafo Ano South) district in Ghana.

**Methods:**

Interviews were conducted with representatives of 32 institutions engaged in promoting malaria prevention and control. The averaging technique proposed by Brown et al. and a two-level multinomial multilevel ordinal logistic regression were used to examine the levels of integration and the factors that explain the differentials.

**Results:**

The results show high disparity in levels of integration amongst institutions in the two districts. Integration was higher in the rural district compared to the urban district. The multivariate analysis revealed that the district effect explained 25% of the variations in integration. The type of institution, level of focus on malaria and source of funding are important predictors of intersectoral integration.

**Conclusion:**

Although not causal, integrated malaria control programmes could be important for improving malaria-related health outcomes in less developed regions as evident from the rapid decline in malaria fatality rates observed in the Ahafo Ano South district. Harmonisation of programmes should be encouraged amongst institutions and the public and private sectors should be motivated to work in partnership.

## Introduction

The Government of Ghana has instituted several policies and programmes aimed at reducing malaria-related morbidity and mortality [1–5]. Although these programmes and policies have resulted in steady improvements in intervention coverage, disease incidence and mortality [6,7], malaria continues to be hyperendemic in all parts of the country and is still the number one cause of death [8,9]. In line with the Abuja declaration, recent strategies and programmes have been aimed at reducing malaria incidence by 75% by 2015 [5,10].

Several factors have been associated with the continuing high incidence of malaria in Ghana. These include poverty, poor health care utilisation and environmental-related factors, amongst others [11,12]. Lack of intersectoral integration in the implementation of malaria control programmes has also been touted as a contributing factor to the continuing high incidence [13]. In this study, we investigate the extent of intersectoral integration in malaria control programmes in two districts – a rural district (Ahafo Ano South) and an urban district (Kumasi Metropolitan Assembly), all in the Ashanti Region of Ghana. We also investigated the factors that are associated with differentials in integration amongst institutions in the two districts. Although a number of studies have been conducted to quantify the level of intersectoral integration in health promotion, the factors associated with the differentials have not been systematically studied.

**Figure 1.**
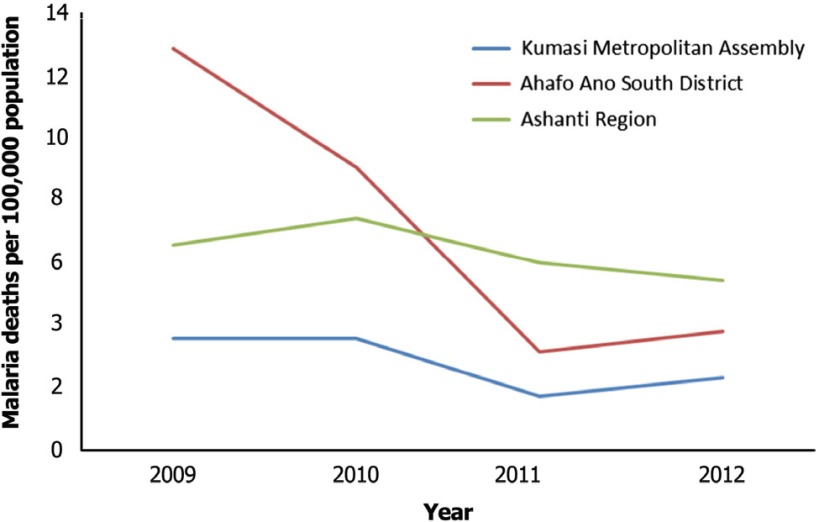
Malaria death rates per 100,000 population. Source of data: The number of malaria deaths was collated from the Ghana District Health Information Management Systems of the Ghana Ministry of Health, Ashanti Region. The projected population for 2009–2012 was computed based on the 2000 and 2010 Ghana Population and Housing Census.

The choice of the two districts is to contrast the integration in a less-developed (rural) to a more-developed (urban) district, noting that the Ashanti region is one of the regions in Ghana with high malaria incidence [14]. The Kumasi Metropolis with a population of 2.03 million [15] is the regional capital of the Ashanti region. It is the most developed district in the region characterised by industries and commercial activities [16,17]. The Ahafo Ano South district with a population of 122,000 [15] is a conglomeration of farming communities engaged mainly in subsistence farming. It is the most poorly developed district in terms of extreme poverty incidence and least resourced with regard to health care provision in the region [16]. Consequentially, Figure 1 shows that malaria death rates are higher in the Ahafo Ano South district when compared to the Kumasi Metropolis. However, there has been a rapid decline in malaria fatality rates in the Ahafo Ano South district, while the rates have stalled in the Kumasi Metropolis and the Ashanti region, in general. From a policy perspective, it is imperative to disaggregate the analysis by the two districts because of their distinct resource endowment, cultural differences, access to health care and progress in malaria control. The evidence from this study could highlight the importance of intersectoral integration for malaria control. The lessons from this study could also be relevant to other regions of the country.

In Ghana, there are many institutions engaged in malaria prevention and control [16]. The integration of institutions is essential for ensuring that programmes which replicate local priorities are sustainable and resources are efficiently used [18]. The success of health policies and programmes does not depend only on the government's efforts but on the efficient coordination of resources amongst institutions, including government and non-governmental agencies (service providers and users, administrators, community members and funders).

A major challenge of intersectoral integration is bringing the many different institutions with different management structures and agendas together in the pursuit of a common goal [18,19]. Nonetheless, the 1986 Haikko Declaration asserted the need for broad intersectoral approaches to promoting primary health care [20]. The importance of intersectoral integration is also affirmed in the ‘Ghana Health Service and Teaching Hospitals Act, 1996, Act 525’. This Act delegates power to the local authorities and health planners and providers to work together in the allocation of resources, planning and implementation of services to reduce inequalities in health delivery [21]. A quarter of a century after the Haikko Declaration, intersectoral integration in health care provision continues to be weak in most parts of the developing world [22] and the factors (which are key to ensuring sustained health) associated with differentials in intersectoral integration have not been systematically studied in most settings, particularly in low and middle-income countries. The findings from this study could aid the targeting of resources for promoting intersectoral integration for health care promotion.

## Data and methods

### Data

The data for the analysis come from interviews conducted with coordinators and representatives of 32 (16 from each district) core institutions engaged in malaria control programmes in the Kumasi Metropolis and Ahafo Ano South district. The selection of institutions was based on the review of literature and discussions with representatives of the Ghana Health Service who oversee the implementation of health policies and programmes in Ghana. The participating institutions were not restricted to the health sector, but included the agriculture, education, environment and economic/finance sectors among others. The types of personnel who were interviewed included service providers, administrators, service users/community members (including local politicians). After identifying all the relevant institutions, a formal letter was sent to solicit their participation in the study. The institutions were invited to select their own representatives. The representatives were mainly the heads of the institutions, deputy heads and/or district coordinating officers. Where more than one representative from an institution was interviewed, the responses were averaged over the number of representatives. Coordinators and representatives from the following organisations were interviewed in the two districts – Malaria Control Programme; Ministry of Agriculture; Ministry of Education; Ministry of Social Welfare; Ministry of Finance and Economic Planning; Planned Parenthood Association of Ghana; Mutual Health Insurance Scheme; Diseases Prevention Control Unit; Health Assessment and Disease Surveillance Unit; Town and Country Planning Department; Community Water & Sanitation Agency; Family Planning & Immunization Unit; National AIDS/STI Control Programme; Chemical Sellers Association, Traditional Healers; and community leaders and local politicians. Ethical clearance was obtained from the Faculty of Social Human Sciences Ethics Committee at the University of Southampton and also the Ghana Ministry of Health. The study was conducted between November and December 2009.

In this study, we adopted the integration indicators developed by Narayan and Shier [23] and adopted by Browne et al. [24] and Ahgren and Axelsson [25]. The indicators are scored on an ordinal scale – 0 = ‘non-awareness’, 1 = ‘awareness’, 2 = ‘communication’, 3 = ‘cooperation’ and 4 = ‘collaboration’. In our study, all the institutions interviewed were aware of each other's programmes, thus the measures of integration are defined as:

**Awareness**: If an institution has knowledge of another institution's malaria control programmes, but does not participate in their activities – classified as very low level of integration in this study.

**Communication**: If an institution has knowledge of another institution's malaria programmes and they only share information on their activities – classified as low level of integration.

**Cooperation**: If an institution has knowledge of another institution's malaria programmes and not only shares information, but also shares ideas to guide and modify their own planning and activities – classified as moderate level of integration.

**Collaboration**: If an institution has knowledge of another institution's malaria control programmes, they share both information and ideas and also jointly plan and modify delivery of service based on mutual consent – classified as high level of integration.

The sixteen institutions in each district were asked to score their level of integration with the remaining institutions in the programme. The responses resulted in 512 observations nested within 32 institutions. Other background information collected from the institutions were type of institution, categorised as ‘government’ or ‘non-government’; level of focus on malaria (high, moderate and low); source of funding (government, donors and other); and main service provided (health, food and environment, education and social services, and health finance).

### Methods

Two methods of analyses were used to examine the level of integration and the factors that explain the differentials. The first was the averaging technique proposed by Brown et al. (2004) which was used to quantify the extent, scope and depth of integration amongst the institutions, both between and within the districts [24]. The second was a two-level multinomial multilevel ordinal logistic regression technique which was used to investigate the factors that explain the differences in the levels of integration [26]. To measure the level of integration, we assume that the sixteen institutions in each district exist as a network and as such successful integration is achieved by how well the members of this network are aware of each others’ activities with regard to malaria control programmes and subsequently communicate, cooperate and collaborate [24].

The responses from each of the institutions were inputted into a matrix. Representatives/coordinators from each of the institutions listed in the first column of the matrix, are asked to score their level of integration with other members of the network, listed in the first row of the matrix. From this matrix, three facets of integration were calculated – group-reported depth of integration, self-reported depth of integration and total-observed depth of integration. The estimated group-reported and self-reported depth of integration scores represents the row and column means, respectively whilst the total-observed depth of integration score is the mean of the mean scores. Group-reported depth of integration measures the degree of integration of an institution as perceived by the members of the network, whilst Self-reported depth of integration measures the depth of integration of the members of the network as perceived by an institution. The total-depth of integration score is the overall perceived level of integration amongst institutions in the network.

We used the *paired Wilcoxon signed-ranked test* to examine if the group-reported and self-reported depth of integration scores for each institution were significantly different at the 5% level of significance [27]. This test was adopted because it is nonparametric, appropriate for ordinal measures and non-random samples, does not assume normality and it is suitable for small sample sizes (*n* = 15 in this case<30). Since each institution scores all other members of the network, it was assumed that there is homogeneity in the responses; therefore the paired test procedure was adopted. The significance of this test will imply that individual members’ perception and the groups’ perception with regard to level of integration are significantly different, which will imply inconsistencies with regard to how members of the network perceive their level of integration. The *Mann–Whitney test*, which is the unpaired alternative of the *Wilcoxon signed-ranked test*, was used to examine if the total-observed depth of integration scores differ significantly between the two districts [27]. The unpaired test was used in this case because the scores from the institutions in the two districts are independent.

In the second part of the analysis, a two-level multinomial multilevel ordered logistic regression technique was used to examine the factors that explain the difference in the levels of integration amongst the institutions. This technique was adopted because the response variable is on an ordinal (ordered) scale and responses are nested within institutions. In order words, 512 responses (at level-one) nested within 32 institutions (at level-two) comprised the hierarchical data structure for the analysis. The response variable is coded 1 if an institution is only aware of another institution's malaria control programmes, 2 if an institution is aware of another institution's programme and they communicate, 3 if an institution is aware of another institution's programme, they communicate and cooperate, and 4 if an institution is aware of another institution's programme, they communicate, cooperate and collaborate.

Suppose *y*_*ij*_ is the response *i* (level-one) from institution *j* (level-two) and the probability of being in response category *s* is denoted by 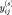, a multinomial generalisation of the proportional odds model with a logit link can be defined for a two-level model with *t* response categories as:
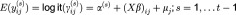

One category of *s* is chosen as the reference category, *X* is the vector of covariates and *β* are the regression coefficients which are the same for each of the response categories and *µ*_*j*_ ∼ *N*(0, *τ*^2^) is the level-two random effect. The significance of the level-two (institutional effect) variance *τ*^2^ would imply that there significant differences in integration amongst the institutions, after accounting for the variables in the model. A three stage modeling process was adopted to investigate how the background characteristics of the institutions explain their level of integration. Model 1 accounted for only the institutional structure of the data, Model 2 included the district (Kumasi Metropolis or Ahafo Ano South) and Model 3 further added the background characteristics of the institutions. At each stage of the model building process the variables that were not significant at *p*<0.05 were discarded. The significance of variables was further tested in the final model. The adaptive quadrature estimation procedure via the Generalised Linear Latent and Mixed Models (*gllamm)* function in STATA version 12 was used to estimate the model parameters [28,29].

### Results

#### Level of intersectoral integration in malaria control

Table 1 shows the estimated integration scores for institutions participating in malaria control programmes in the Kumasi Metropolis and the Ahafo Ano South district. Since all the institutions in the survey are aware of each other's programmes, the scores ranged between 1.0 and 4.0. The higher the estimated score the higher the level of integration. The scores were categorised (in a rounded form) to match the ordinal scale measures that were adopted. Scores ranging from 1.0 to 1.4 were classified as ‘very low’ level of integration and are deemed to reflect institutions that are only aware or are perceived to be only aware of other institutions programmes. Those between 1.5 and 2.4 were classified as ‘low’ level of integration, reflecting institutions that are aware or are perceived to be aware of other institutions programmes and communicate but do not cooperate or collaborate in their activities. Scores ranging between 2.5 and 3.4 were classified as ‘moderate’ level of integration, and reflect institutions that are or are perceived to be aware of other institutions programmes, communicate and cooperate but do not collaborate. Finally, scores between 3.5 and 4.0 were classified as ‘high level of integration’, reflecting institutions that collaborate or are perceived to collaborate with other institutions. The estimated group-reported and self-reported depth of integration scores shows high degree of disparity in the levels of integration as perceived by the institutions within the network.

The group-reported depth of integration scores for the Kumasi Metropolis range from 1.6 for the Mutual Health Insurance Scheme to 3.2 for community leaders/local politicians, while the self-reported depth of integration scores range from 1.5 for Traditional Healers to 3.2 for the National AIDS/STI Control Programme. This shows that there are members of the network who are perceived to exhibit low level integration. For the Ahafo Ano South, the group-reported depth of integration scores range from 2.3 for the Ministry of Social Welfare to 3.9 for community leaders/local politicians, while the self-reported depth of integration scores range from 2.2 for Traditional Healers to 4.0 for community leaders/local politicians. This indicates that there is consensus that community leaders are highly involved in malaria control programmes in the Ahafo Ano South district, reflecting their roles and importance.

The overall level of integration as measured by the total-observed depth of integration scores was 2.9 for the Ahafo Ano South district and 2.4 for the Kumasi Metropolis. This indicates that there is lower level of integration amongst institution in the Kumasi Metropolis, whilst there is moderate level of integration amongst institutions in the Ahafo Ano South district. This shows that majority of institutions in the Kumasi Metropolis are aware of each other's activities; they communicate but do not cooperate or collaborate, whereas for Ahafo Ano South district the institutions are aware, they communicate and cooperate but do not collaborate.

The tests of significant differences in the scores revealed that for both districts the differences between the group-reported and self-reported depth of integration scores were not statistically significant. This shows that the way the network members perceive themselves with regard to their level of integration are not statistically different, demonstrating that individual members’ perception and the groups’ perception are similar. However, the difference between the total-observed depth of integration scores for the two districts were statistically significant (*p* < 0.05). This reveals that the level of integration amongst institutions engaged in malaria control programmes in the two districts are considerably different. In other words, integration amongst institutions in the Ahafo Ano South district is higher when compared to those in the Kumasi Metropolis.

The estimated group-reported depth of integration scores for the Kumasi Metropolis reveals that one-half of the network members were perceived by other members to exhibit low level of integration, while the other half were perceived to exhibit moderate level of integration (Table 1). For the Ahafo Ano South, a quarter of the institutions were perceived by other members to exhibit low level of integration, two-thirds were perceived to show moderate level of integration, while the remaining institutions were perceived to have high level of integration (Table 1).

#### Factors associated with differentials in the levels of intersectoral integration

The estimated odds ratios and their corresponding 95% confidence intervals from the fitted two-level multinomial multilevel ordinal logistic regression models are presented in Table 2. The results also shows the estimated variance of the random effects term (institutional level variance) attributable to the unexplained differences in levels of integration amongst institutions after accounting for the predictors in the model. The significant institutional level variance shown in Table 2 (Model 1) reveals that, without accounting for any predictors in the regression model, there exist significant differences in the level of integration amongst institutions in the network. Model 2 which accounts for district, shows that 25% of the variation is explained by the district effect. Accounting for the type of institution, level of focus on malaria and main source of funding explained 98.7% of the remaining variance. This clearly indicates that the extent of integration amongst institutions which are engaged in malaria control programmes in the two districts is highly dependent on these three factors.

The estimated odds ratios presented in Table 2 reveal that the odds (odds ratio = 0.09, 95% confidence interval = 0.05, 0.14) of institutions engaged in malaria control programmes in the two districts being aware of other institutions programme and not communicating, cooperating and collaborating is low. They are 58% less likely to be aware and communicate without cooperating or collaborating. However, they are 2.31 times more likely to be aware, communicate and cooperate and not collaborate with other institutions. The multivariate analysis shows a significant difference in levels of integration between the two districts, even after accounting for the institutional effect and other background characteristics. This confirms the independent effect of district (rural versus urban) on the level of integration. Institutions in the Ahafo Ano South district are 61% more likely to integrate compared to those in the Kumasi Metropolis, further confirming the differentials identified in the bivariate analysis.

With regard to type of institution, non-governmental institutions are 11.42 times more likely to integrate when compared to government institutions. Institutions with a high focus on malaria are 55% more likely to integrate when compared to those with moderate focus on malaria and 68% more likely when compared to those with a low focus on malaria. The results further show that institutions whose main source of funding is from donors are two times more likely to integrate when compared to those funded mainly by government. The type of services provided by the institutions does not significantly influence their level of integration after accounting for the predictors in the model. We also tested for plausible interaction effects, however none were significant. This is not surprising given that the main effects explain almost all the variations in level of integration.

## Discussions and conclusions

The causes of malaria are complex and multifaceted and as such its prevention and control call for intersectoral integration to ensure efficient use of resources and implementation of sustainable programmes at the grassroots level. This paper set out to investigate the extent of integration amongst institutions engaged in malaria control programmes in the Kumasi Metropolis and the Ahafo Ano South district in the Ashanti region of Ghana. The paper also examined the factors that contribute to variations in the levels of integration between institutions. Understanding the factors that account for differentials in integration is essential for setting out strategies for promoting intersectoral programmes and ensuring efficient use of resources. It is important to note that this study focused on two districts of Ghana and as such does not allow for generalisation of the results nationally. Nonetheless, the research sets the scene for a national level study that could aid understand harmonisation of programmes.

The results show low level of integration amongst institutions in the urban district (Kumasi Metropolitan Assembly), whilst there is moderate level of integration amongst institutions in the rural district (Ahafo Ano South). Statistical tests revealed that the level of integration is significantly higher amongst institution in the less developed Ahafo Ano South district (group-reported depth of integration score = 2.9) when compared to the more developed Kumasi Metropolis (group-reported depth of integration score = 2.4). Inferring from Figure 1, although the Ahafo Ano South district is poorly developed, less resource endowed and with inadequate access to health care, compared to the Kumasi Metropolis, it has made substantial progress in malaria fatality rates, whilst the rates have stalled in the Kumasi Metropolis and the Ashanti region in general. This gives a positive indication though not of causation that integrated malaria control programmes could be important in improving malaria related health outcomes, particularly in less developed regions.

The difference in the level of integration between the two districts is in conformity with the argument that levels of social capital influence the effectiveness of social institutions. Thus, those who adhere to this view would argue that the more developed Kumasi Metropolis, which is well resourced with improved communication facilities, road networks, transportation facilities and human capital, would display higher levels of institutional integration. Nonetheless, even in a setting characterised by high levels of social capital, institutions are less likely to function as a coordinated system if there are differences in organisational agenda, expectations and lack of trust [30–32].

Integration has often been measured from the perspective of professionals [25]. However, the perspectives of service users are important for understanding how programmes replicate local priorities and needs [25,33]. In this regard, recent models have proposed measures that capture the extent of cohesion between users and professional and also between professionals [25]. In reference to this study, the group-reported depth of integration score for community leaders/local politicians for the Ahafo Ano South district was 3.9, whilst the self-reported depth of integration score was 4.0 compared to 3.2 and 2.9, respectively for the Kumasi Metropolis. This clearly demonstrates that integrated programmes involving active participation of local communities are important (although not causal) for improving malaria-related health outcomes. This further substantiates the fact that robust measures of intersectoral integration should capture users’ perspectives [25].

The group-reported depth of integration scores show that institutions within both districts are less likely to collaborate in the implementation of programmes. Although, there is positive indication of integrated programmes in the Ahafo Ano South district, the lack of collaboration could result in the loss of the strategic benefit of sharing of resources, transfer of knowledge and synchronisation of activities between the partners [34,35]. Furthermore, the absence of collaboration can cause institutions to be in a situation of having little or no major control on policy implementation [35]. Thus, although the institutions interact, there exist institutional or philosophical barriers which make integrative coordination in planning and execution of activities more difficult to be achieved [35,36].

The problem of malaria control needs policy and programme efforts that contribute positively to the efforts of all sectors. At best, it necessitates working in a more strategic and coordinated manner with all players adopting policies and programmes that promote a sector-wide approach to malaria control. It also requires an efficient set of communication linkages among all stakeholders to maintain and sustain such comprehensive process of intervention programmes. In the policy context, the fact that there are many stakeholders involved, from both the public and private sectors, demands an integrative policy arrangements of which structural issues of management and governance are critical ingredients to the development of such integration.

## Reviewers

**Hukum Chandra**, Senior Scientist, Indian Agricultural Statistics Research Institute, New Delhi, India.

**Mario Cruz-Peñate**, MD, MPH, Regional Advisor on Primary Health Care, Pan American Health Organization PAHO/WHO, Washington DC, USA.

**Kathy Dennill**, Director, Kedibone Health System Consultants, Banbury, Randburg, South Africa.

## Figures and Tables

**Table 1.**
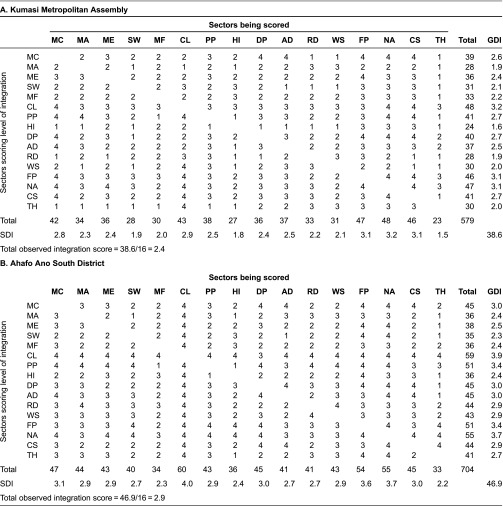
Level of integration among institutions participating in malaria control programmes.

GDI – Group reported Depth of Integration; SDI – Self-reported Depth of Integration; MC – Malaria Control Programme; MA – Ministry of Agriculture; ME – Ministry of Education; SW – Ministry of Social Welfare; MF – Ministry of Finance and Economic Planning; CL – Community leaders/local politicians; PP – Planned Parenthood Association of Ghana; HI – Mutual Health Insurance Scheme; DP – Diseases Prevention Control; AD – Health Assessment and Disease Surveillance Unit; RD – Town and Country Planning Dept; WS – Community Water & Sanitation Agency; FP – Family Planning & Immunisation Programme; NA – National AIDS/STI Control Programme; CS – Chemical Seller Association; TH - Traditional Healers

Note: Perceived Scope of integration and self-reported scope of integration is 100% for both districts because all the sectors are aware of each other.

**Table 2.**
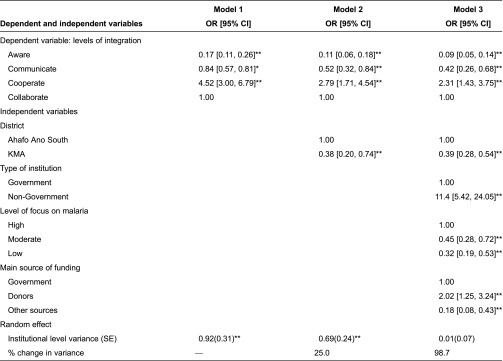
Estimated odds ratios, their 95% confidence intervals and random effect variance from a two-level multinomial multilevel ordinal logistic regression.

OR – odds ratio; CI – confidence intervals; SE – standard error.

*p<0.05; **p<0.01.
